# Towards understanding various influences on mass balance of the Hoksar Glacier in the Upper Indus Basin using observations

**DOI:** 10.1038/s41598-022-20033-w

**Published:** 2022-09-19

**Authors:** Shakil Ahmad Romshoo, Khalid Omar Murtaza, Tariq Abdullah

**Affiliations:** Department of Geoinformatics, University of Kashmir, Hazratbal, Srinagar, India

**Keywords:** Climate sciences, Climate change, Cryospheric science

## Abstract

Mass balance is a good indicator of glacier health and sensitivity to climate change. The debris-covered Hoksar Glacier (HG) in the Upper Indus Basin (UIB) was studied using direct and geodetic mass balances. During the 5-year period from 2013 to 2018, the glacier’s mean in situ mass balance (MB) was − 0.95 ± 0.39 m w.e. a^−1^. Similarly, the glacier’s mean geodetic MB from 2000 to 2012 was − 1.20 ± 0.35 m w.e. a^−1^. The continuously negative MB observations indicated that the HG is losing mass at a higher rate than several other Himalayan glaciers. The glacier showed increased mass loss with increasing altitude, in contrast to the typical decreasing MB with increasing elevation, due to the existence of thick debris cover in the ablation zone, which thins out regularly towards the accumulation zone. Rising temperatures, depleting snowfall and increasing black carbon concentration in the region, indicators of climatic change, have all contributed to the increased mass loss of the HG. During the lean period, when glacier melt contributes significantly to streamflow, the mass loss of glaciers has had a considerable impact on streamflow. Water availability for food, energy, and other essential economic sectors would be adversely affected, if, glaciers in the region continued to lose mass due to climatic change. However, long-term MB and hydro-meteorological observations are required to gain a better understanding of glacier recession in the region as climate changes in the UIB.

## Introduction

Outside the polar regions referred to as ‘the third pole’, the Himalaya has favorable climatic and topographic setting for hosting a large number of glaciers. The snow and glacier melt from the third pole contributes significantly to streamflow of the major Himalayan rivers, sustaining irrigation, hydropower, and a variety of other water-related activities in the region and beyond^1,2^. Therefore, research into a vast number of glaciers in the region continues to focus on their status, dynamics, hydrological significance and their responses to climate change and environmental pollution^3–5^. The mass balance of Indian Himalayan glaciers is largely negative with a recent increase in mass loss^6,7^. Because most glaciers in the Himalaya are small, they have a higher rate of mass loss. Several studies have found spatial variation in glacier mass loss across the Himalaya^4,8–10^. The Kashmir and the Lahaul-Spiti glaciers in the western Himalaya have experienced the highest relative mass loss^8,11^. The glaciers in the central and eastern Himalaya have comparatively experienced lower rates of mass loss^12,13^. However, to a few studies, glaciers in the Karakoram region are more or less in a stable state and a few glaciers are even advancing or surging^14,15^.

Glaciers in the Upper Indus Basin (UIB) are important because glacier-melt accounts for a major component of the streamflow and supports agriculture, water supply, energy generation and tourism, all of which are important sectors of the economy in the basin^16,17^. Glaciers in the UIB, like those elsewhere in the Himalaya, have experienced significant recession and mass loss in recent decades^4,18^. Several studies have shown that glaciers in the UIB have receded since the end of the Little Ice Age^19,20^. Recent research has also revealed that some of the glaciers in the basin may be melting at a faster rate in recent decades^17,21^.

Glacier MB is the most reliable metric for assessing the health of a glacier and its response to climate change^7,22,23^. However, studying glacier MB in the Himalaya is difficult due to a combination of adverse factors such as rugged topography, remoteness, security concerns, logistical constraints, and a lack of hydro-meteorological observations^24,25^. As a result, there are only a few mass balance records for Himalayan glaciers^6^. Despite a clear indication of climate change impacts on glaciers, no other glacier in the Kashmir Himalaya has been studied for mass wastage using field observations except the Nehnar Glacier in the Jhelum sub-basin, which has an MB record of 9 years from 1975^26^. Mass balance studies of two more glaciers in the Jhelum, the Kolahoi and Sheshram Glaciers were conducted for a single year and then discontinued before being abandoned^27^, however authenticity of the data has been questioned^28^. But, a few remotely sensed mass balance studies for a few glaciers have been carried out^8,18^.

An increase in air temperature, change in the form and phase of precipitation^29,30^ and the increasing emission of black carbon^31^ are all factors and processes that contribute to glacier mass loss in the Himalaya. The growing anthropogenic effects such as emission of black carbon, whose concentration significantly contributes to atmospheric warming^32,33^ and its deposition on glaciers, which reduces albedo and causes absorption of solar radiation prompting enhanced glacier-melting. Black carbon has been found to be an important factor that influences the melting of Himalayan glaciers in a number of studies^5,34–36^. Glaciers across the Himalaya have been negatively influenced by climate change^37^ and the observed mass loss, if it continues, will have a considerable impact on regional hydrology, and water supplies in the downstream areas^38^. The Himalayan glaciers must be monitored and assessed on a regular basis in order to understand their health and evolution in the face of changing climate. The knowledge gained from cryosphere research shall inform the national policymaking for sustainable water resources management in the Himalayan catchments, which are home to more than a billion people in south Asia^39^.

The current study investigated annual field-based glaciological mass balance measurements from 2013 to 2018 and geodetic mass balance utilizing digital elevation data from 2000 to 2012 to better understand glacial mass wasting of the HG. The observed mass balance estimations were compared to the climatic parameters that influence glacier mass balance. The findings are expected to provide a better understanding of the health and evolution of glaciers in the UIB in the recent past.

## The Hoksar Glacier

**Figure 1:**
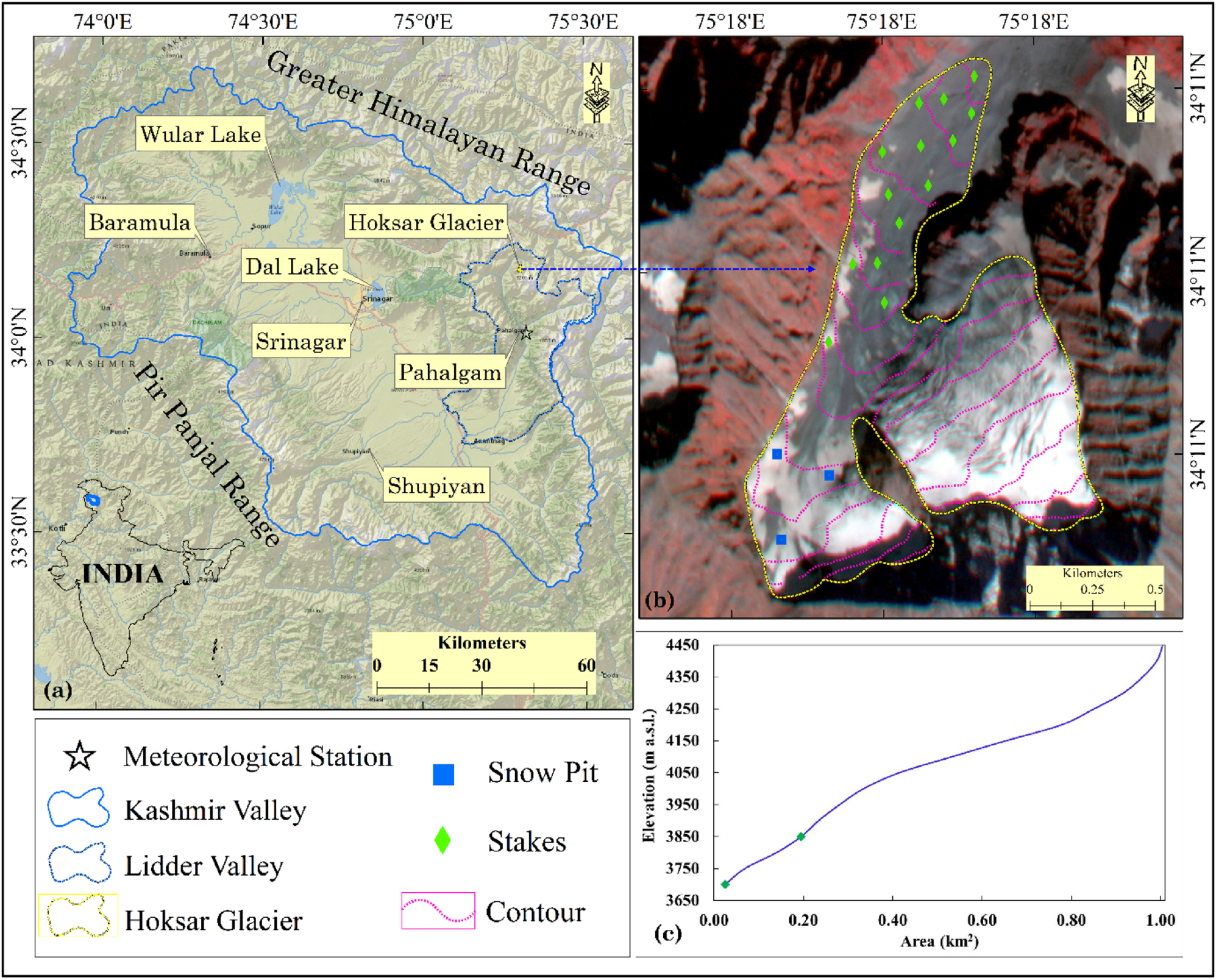
(**a**) Map showing the location of the Kashmir valley (blue color boundary) with respect to India country map, Lidder valley (dotted dark blue color) and other key landmarks including the Hoksar Glacier (dotted yellow color) and location of the meteorological station at Pahalgam (star). (**b**) Satellite imagery of the Hoksar Glacier showing the contour lines generated from SRTM DEM (dotted pink color), distribution of debris-cover, location of stakes (green diamond boxes), accumulation pits (blue square boxes), and (**c**) Cumulative hypsometric curve of the HG, two green dots depict the glacier area with a thick debris-cover. Maps (**a**,**b**) were generated in ArcGIS version 10.4.1 (https://www.esri.com) by utilizing the imageries provided by National Geographic, Esri and Planet Labs dated 23rd Sep 2019, respectively, (**c**) hypsometric curve was plotted using Microsoft office excel 2019.

The Hoksar Glacier (HG), a small valley type glacier with an area of ~ 1 km^2^, is located between 34° 10′–34° 11′ N and 75° 17′–75° 18′ E on the northern slopes of the Greater Himalayan range in the Jhelum sub-basin, a major tributary of the Indus (Fig. 1). The HG has a mean elevation of ~ 4000 m a.s.l, is oriented north-west with a slope of 20°–30°, and drains into the Lidder river, which is one of the tributaries of the Jhelum in the UIB^40^. The HG’s current snout elevation is 3680 m. The central flowline length of the glacier measures ~ 2.2 km with width ranging from ~ 300 to 1200 m. The glacier is covered with debris, which currently covers around 80% of the ablation area, with varying debris thickness made up primarily of rock fragments measuring a few millimeters to tens of centimeters and large boulders exceeding half a meter in size. Figure S1 depicts a few of the glacio-geomorphic features present in the Hoksar glacial valley, which indicate past extents of the HG.

## Materials and methods

### Datasets used

Several datasets were used to assess the mass change of the HG, which are briefly discussed below and listed in Table 1.

**Table 1:** Description of the datasets used in this study.

Dataset	Spatial/temporal resolution	Acquisition date	Source
SRTM DEM	90 m	11–22 Feb 2000	http://srtm.csi.cgiar.org
TanDEM-X DEM	90 m	December, 2010–Janurary, 2015	https://download.geoservice.dlr.de/TDM90/
**Climate data**			
Temperature and precipitation	Station data/daily data	1980–2018	IMD Pahalgam
**Non-climatic data**			
Black carbon	0.5° × 0.625°	1980–2017	MEERA v2
Black carbon	5 min	21–24 Sep 2013	In-situ

### Remote sensing data

Geodetic mass balance of the HG was estimated using two Digital Elevation Models (DEMs), the Shuttle Radar Topography Mission (SRTM) DEM and TanDEM-X DEM, from 2000 to 2012. The SRTM DEM was acquired in two frequencies (X-band and C-band) between 11 and 22, February 2000. In this research, the non-void filled version of the SRTM DEM with a spatial resolution of 90 m was used. The TanDEM-X mission is a TerraSAR-X-Add-on for Digital Elevation Measurements, which was acquired between 2010 and 2015 and was time-stamped as 2012 in this study. The TanDEM-X 90 m DEM, which was used in this study, is a product variant of the global DEM with vertical accuracy up to 2 m^41^.

### Climatic data

Climate data (1980–2018), namely daily precipitation and temperature, were obtained from the nearest Indian Meteorological Department (IMD) observatory located at Pahalgam in the Lidder valley, which is approximately 19 km away (aerial distance) from the glacier site (Fig. 1), and were used to determine the trends in the time series during the observation period.

### Black carbon data

The portable AE-42 Athalometer (Magee Sci., Inc., Berkeley, CA, USA) ^33^ was used to measure black carbon (BC) concentration on the ablation zone of the HG during one of the field expeditions from 21 to 24 September 2013 for roughly 5–7 h every day. Furthermore, the long-term variation in BC concentration in the Lidder valley was determined using the area-averaged BC concentration from 1980 to 2017, which was obtained from the Modern-Era Retrospective Analysis for Research and Applications (MERRA).

### Field-observed data

The glacier melt and accumulation measurements were carried out during the observation period at different altitudinal ranges of the HG using stakes and snow pit/probes, respectively. We also used the Differential Global Positioning System (DGPS) to monitor the snout position and altitude over the course of the study from 2013 to 2018.

### Methods

The purpose of the research was to use diverse approaches to understand and quantify the mass wastage of the HG and the reasons behind it. The methodology adopted to achieve the objective of the present study is described in the following sub-sections.

### Glacier mass balance approaches

Glace mass balance is calculated by several methods, viz., glaciological^42^, geodetic^43^, AAR^44^, hydrological^45^, empirical model^46^ and gravitational^47^. However, in this study, we used glaciological, and geodetic methods to calculate the mass balance of the HG to gain insight into the recent glacier mass changes and the controlling factors thereof.

### Glaciological method

The glaciological mass balance method is an in-situ measurement of accumulation and ablation over the entire glacier during a hydrological year that provides a direct indication of mass changes^42^. In this study, glacier melting was measured using a network of bamboo stakes anchored in the ablation zone of the HG. Whereas, snow accumulation was accounted for using several snow pits dug into the accumulation zone of the glacier complemented by a number of snow probes. The annual glacier-wide mass balance, *b*_*n*_ is calculated as follows:

1 $${b}_{n}=\frac{1}{S}\sum {b}_{i}{s}_{i}$$

where *b*_*i*_ is the MB of the altitudinal area (*s*_*i*_) obtained from the corresponding stake readings or net accumulation measurements and *S* is the total glacier area.

Mass balance measurements on the HG began in the year 2013 with the objective of creating a long-term mass balance dataset for the glacier to investigate glacier-climate interactions and the influence of other controlling factors on glacier response like debris, etc. The mass balance was calculated using 14 stakes and 3 snow pits (supplemented by several snow probes), positioned over the ablation and accumulation zones respectively (Fig. 1) and the glacier-wide mass balance was obtained by applying an integration over the entire glacier surface. The number of stakes remained constant throughout the observation, however, the number of accumulation pits increased from one in 2013 to 2014 to three in subsequent years. The number of snow pits is limited by the complexity of the accumulation area as well as other logistical considerations. For snow depth and density measurements, 3–5 snow pits, supplemented by snow probes, are usually recommended^48^. However, in this study, a large number of snow probes placed across the accumulation zone augmented the data from the snow pits. All three snow pits were located on the right side of the accumulation area. Though, there should have been at least one snow pit on the left side of the accumulation area. It was not possible to locate one there due to the complex terrain, crevasses and logistical constraints. Using DGPS, the precise location of each stake and snow pit, and snow probe was determined. The hydrological year extends from October to September in the Kashmir Himalaya. The mass balance measurements were accordingly carried out towards the end of September or the beginning of October every year during the annual glacier field expeditions from 2013 to 2018. Steam-driven Heucke ice drill was used to drill 5–6 m deep ablation stakes^49^. For volume to mass conversion, the average density of the annual layer of snow/firn and ice was measured altitude-wise from the accumulation area (550 kg m^−3^) and ice density of 900 kg m^−3^ for the ablation zone of the glacier was used from literature^51^.

To assess the influence of non-climatic factors on glacier mass balance such as debris-cover and thickness variability, the mass change measured at stakes located in the clean and debris-covered portions of the glacier were analyzed separately. The supra-glacial debris extents were mapped from the satellite images and validated on the ground (110 sites), while the debris thickness was measured manually on the ground around each stake (point specific) and averaged over the observation period.

### Geodetic method

Geodetic mass balance approach involves comparing two glacier surface elevation datasets from topographic maps or digital elevation models to determine the volume change over the considered time span. By applying the density of snow/ice at different parts of the glacier, the volume change can be converted into mass change^52^. The geodetic mass balance, ${B}_{geo}$ within the time period ∆t is calculated by multiplying the glacier volume change (∆V) by the mean estimated glacier density $(\rho )$ as follows:

2 $${B}_{geo}=\Delta V\times \rho $$

The participating DEMs in this study were corrected for vertical and horizontal off-sets using the universal co-registration algorithm^53^. The algorithm employs a slope normalized cosine relationship between aspect and elevation change to minimize the offsets as follows:

3 $$\frac{dH}{\mathrm{tan}(\alpha )}=a\cdot \mathrm{cos}\left(b-\Psi \right)+C$$

4 $$C=\frac{\overline{dH}}{\overline{\alpha } }$$

where α is slope; Ψ is glacier aspect; and the variables a, b, and c are the magnitude, direction, and mean bias, respectively. *dH* and $\overline{dH }$ is elevation difference and overall elevation bias, respectively. The minimization process was repeated till either the magnitude of shift was < 0.5 m or the normalized median absolute difference (NMAD) on off-glacier terrain was < 5% than the previous pass^53^. The voids in the DEMs were filled using the Natural Neighbour (NN) algorithm^8^.

The DEMs were corrected for radar penetration bias before generating the DEM difference map at a pixel level. The relative penetration bias was calculated as a function of altitude^8,11^. Hoksar Glacier boundary was used to calculate the mean glacier elevation changes between 2000 and 2012 and the volume changes thereof. Using the density conversion factor of 850 kg m^−3^, the volume changes were then converted into glacier mass changes^54^.

### Equilibrium line altitude (ELA) changes

ELA is the average altitude at which accumulation exactly balances ablation over a period of 1 year. ELA of the HG was estimated each year using regression analysis of the altitudinal mass balance measurements taken throughout the observation period^55^. ELA is a good short-term indicator of glacier health^56,57^ and it has been used to calculate the mass balance of a glacier for which historical data is available^44^. The ELA changes were used as corroborating evidence for the observed mass losses of the HG.

### Climate data analysis

Temperature and precipitation records were analyzed from 1980 to 2018 to determine the trends and demonstrate a link between changing climatic variables and glacier mass loss. The daily climate data used in this study were obtained from the Indian Metrological Department (IMD) Pahalgam station which is the nearest observatory to the glacier. The data was checked for quality, consistency, and gaps. It is important to note that the data gaps were minimal-less than 1%. Therefore, we do not need to correct the data. The significance of the trends in the meteorological parameters was determined using the Mann–Kendall statistical nonparametric test^58,59^. Temperature lapse rates (TLRs) are commonly used to demonstrate the relationship between temperature and glacier mass loss in absence of the meteorological observations near or on the surface of a glacier^60^. The temporally and spatially variable TLRs used in this study were adopted after Romshoo and others^61^. At the HG altitude, the extrapolated temperatures indicated mean minimum and maximum temperatures of − 4.51 °C and − 1.42 °C, respectively.

### Black carbon data analysis

The AE-31 series Aethalometer, used in this study, records optical measurements at seven different wavelengths ranging from 370 to 950 nm^33^. BC concentrations were measured at 5-min intervals. The data was retrieved and analyzed using a spreadsheet program to investigate BC patterns at the glacier site during September, 2013 field expedition. Furthermore, trend analysis of the area-averaged MERRA reanalysis of BC concentration data from 1980 to 2017 was used to determine the changes in BC concentration in the Lidder valley. We removed a few outliers observed in the MERRA time series data by averaging data of the 3 years preceding and succeeding the outlier.

## Accuracy estimation

### Glaciological mass balance

The accuracy of the glaciological mass balance estimations depends on various factors, which include the precise measurement of ice loss at each stake location, the spatial averaging of the measurements over the entire glacier, the accuracy of the ice density measurements and the representativeness of stakes and accumulation pits^62–64^. To account for the uncertainty caused by the extrapolation of mass balance in the accumulation zone, we used three different approaches including using a single constant accumulation value for all the glacier elevation bands in the accumulation zone, a linear gradient in accumulation values and an inverse gradient in the highest two elevation bands of the accumulation zone to account for the annual accumulation following Kenzhebaev et al.^65^. Based on the three techniques, the standard deviation in the accumulation was interpreted as the uncertainty of MB in the accumulation zone^65^. For the ablation zone, the MB uncertainty was calculated from one-sigma confidence intervals of the regression coefficients^66^. Based on the accumulation and ablation uncertainties, we calculate an annual glacier-wide mass balance uncertainty of ± 0.39 m w.e. a^−1^ for the HG. The mass balance uncertainty estimates of this study are consistent with those of earlier studies conducted in the Himalaya^67,68^.

### Geodetic mass balance

The uncertainties of the geodetic mass balance estimates arise due to several factors including the uncertainty due to DEM differencing, radar signal penetration, uncertainty due to void filling of the source DEMs, delineating glacier outlines, and mass conversion using a single value for the ice density. All these factors were considered for the uncertainty assessment following the methodology of Huber et al.^69^, with an additional term to account for the radar penetration error following Abdullah et al.^8^, assuming that all the errors are uncorrelated and random. The uncertainties analysis of geodetic mass balance is discussed in detail in Supplementary Sect. 1.

## Results and discussion

### Glaciological mass balance

**Figure 2:**
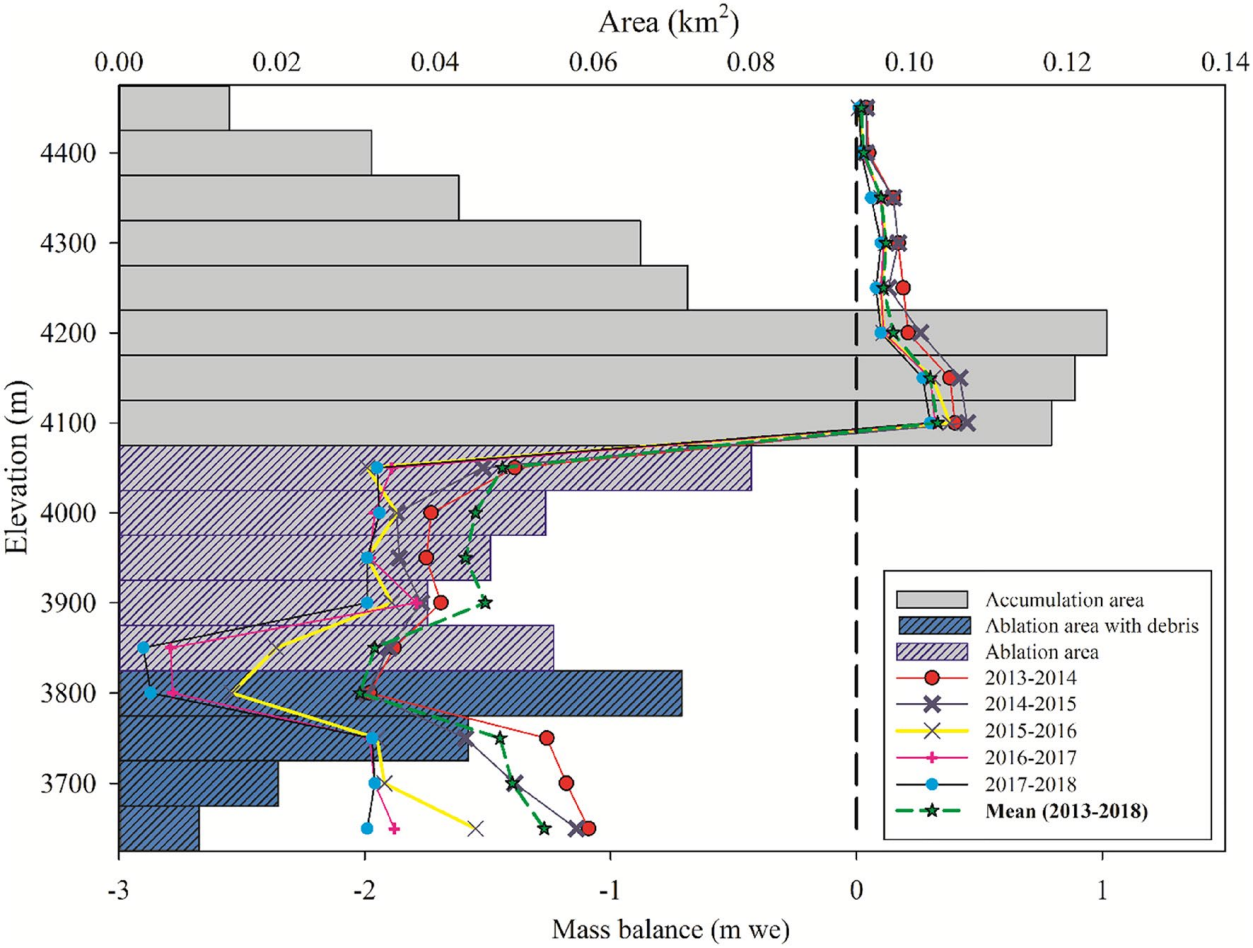
Specific mass balance of the Hoksar Glacier w.r.t. elevation during 2013–2018 derived from the spatially averaged measurements over the given altitudinal band.

Field-based glaciological mass balance measurements of the HG between 2013 and 2018 revealed a significant and continuous mass loss with a 5-year mean mass balance of − 0.95 ± 0.39 m w.e.a^−1^ (Fig. 2; Fig. S2). Mass balance vs elevation of the HG is shown in Fig. 2 (2013–2018). However, the measured mass loss shows significant interannual variability (Table 2) with average annual mass balances of − 0.72 ± 0.39, − 0.78 ± 0.39, − 1.01 ± 0.39, − 1.14 ± 0.39 and − 1.11 ± 0.39 m w.e.a^−1^ across the five hydrological years from 2013 to 2018. In recent years, there has been a higher rate of mass loss with a consistently increasing trend in mass wastage from 2013 to 2018 (Fig. S2). The higher positive mass balance at the 4100–4200 elevation zone is attributed to the fact that this elevation zone has relatively steeper slopes and is situated around the glacier ELA and as a result, the zone witnesses more mass turnover from the accumulation zone. Besides, at times there is additional snow accumulation from the frequent snow avalanche observed in this zone due to its peculiar geomorphology. Therefore, due to these two reasons, there is a relatively higher positive mass balance observed in this elevation band. Furthermore, the hump at 3734 m in Fig. S2 may be attributable in part to the existence of thick debris thickness at the left side of the flowline, and in part to the extra mass turnover from snow avalanches, which is a common occurrence on the eastern flank of the glacier. As a result, for the entire observation period, the mass loss is nearly the same at the spot.

**Table 2:** Mass balance, and ELA changes of the Hoksar Glacier.

Glaciological mass balance	(m w.e. a^−1^)	ELA (m)
2013–2014	− 0.72 ± 0.39	4022
2014–2015	− 0.78 ± 0.39	4031
2015–2016	− 1.01 ± 0.39	4053
2016–2017	− 1.14 ± 0.39	4059
2017–2018	− 1.11 ± 0.39	4062
Average	− 0.95 ± 0.39	Mean ELA = 4045

Geodetic mass balance	(m w.e.)	
2000–2012	− 1.20 ± 0.35 a^−1^	

Supplementary Table S1 shows field-based mass balance data for other Himalayan glaciers, which indicates an overall negative mass balance trend for the glaciers analyzed. Studies of the in-situ mass balance in the Indian Himalaya have indicated a negative but fluctuating trend in the rate of mass loss (Table S1). The mean specific balance of the Indian Himalayan glaciers varies from − 0.24 to − 1.43 m w.e. a^−1^. Recent studies also suggest that glacial mass loss in the region has accelerated in the region over the past few decades, with some variance, primarily due to glacier geometry, debris thickness, and local climatic variability^7,70^. It was further observed that the HG is among the top three glaciers in the Indian Himalaya that has consistently shown a higher rate of mass loss (Table S1).

### Debris-cover influences on the observed mass loss

**Figure 3:**
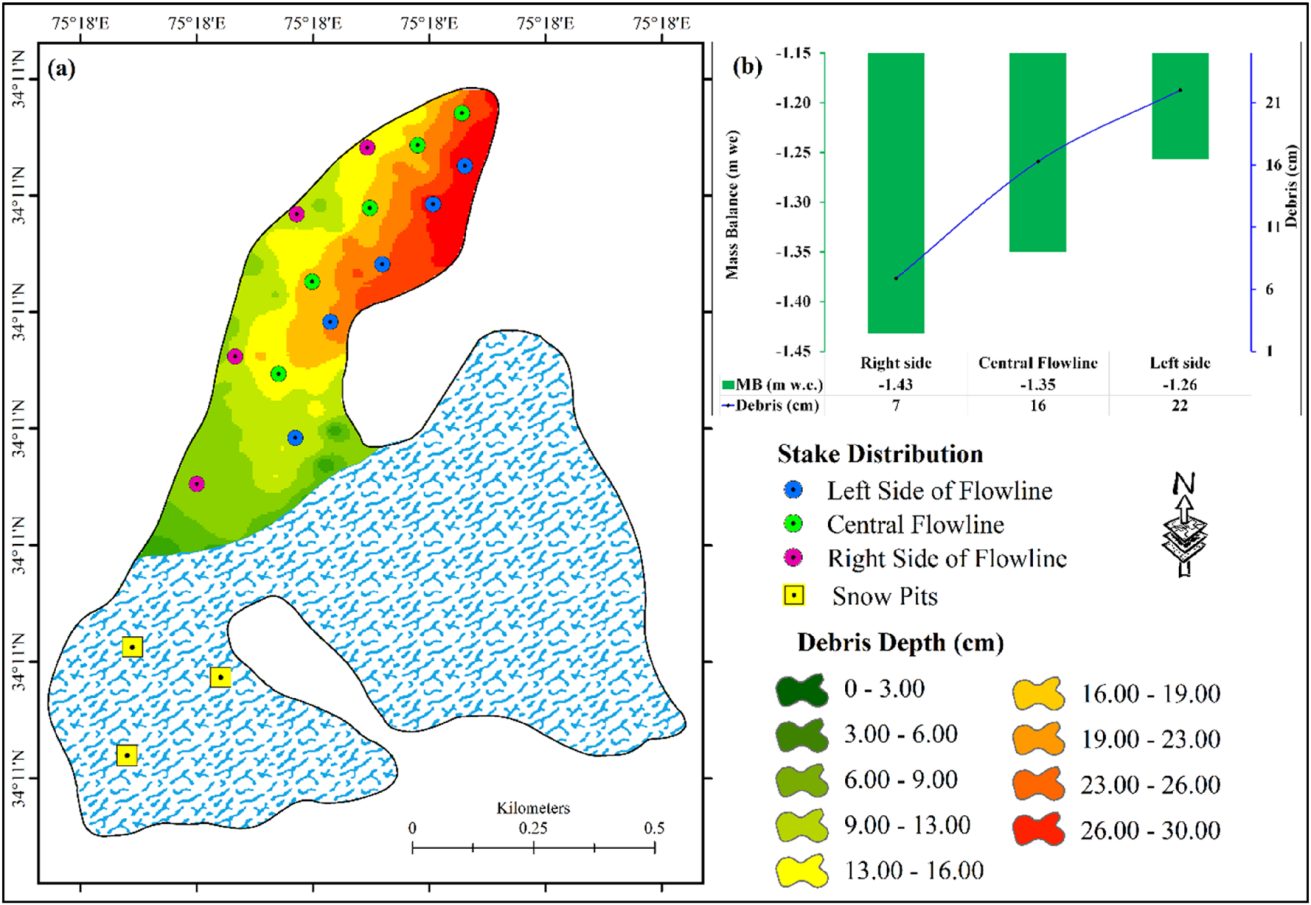
(**a**) Interpolated debris thickness map generated from the *In-situ* measurements, and (**b**) impact of debris thickness on mass loss of the Hoksar Glacier. Stakes when viewed facing the snout were categorized into stakes along the central flowline, stakes on the right side of the central flowline, and stakes on the left side of the central flowline.

During the various annual field expeditions to the Hoksar Glacier from 2013 to 2018, and also corroborated by recent satellite data analysis^20^, it was observed that ~ 80% of the HG ablation zone is covered by debris of varying thickness, ranging from rock fragments as small as one centimetre to boulders as large as 60 cm in size (Fig. 3a). The debris-cover for the glacier was mapped manually from satellite images using the visual image interpretation technique. Furthermore, since the glacier size is relatively small, we were able to extensively validated the remotely-sensed debris cover area in the field during annual glacier field expeditions to the HG. The debris-cover thickness was manually measured in the field using a metal scale. The majority of the debris cover on the glacier mostly comes from the weathered material falling from the steep valley slopes on either side of the glacier valley, with a less amount coming from aeolian material deposited on the glacier surface.

The impact of the non-climatic factors such as debris-cover on the glacier mass changes was assessed independently for stakes along the central flowline, the right side of the central flowline when viewed from the snout, and on the left side of the central flowline. From the analyses of the data (Fig. 3b), it is found that the stakes on the right side of the central flowline have a higher annual mass loss of − 1.43 ± 0.39 m w.e., the stakes along the central flowline have a mass loss of − 1.35 ± 0.39 m w.e. and the stakes on the left side of the central flowline have a comparatively lower mass loss of − 1.26 ± 0.39 m w.e. The presence of a thin debris-cover of 6.9 cm on the right side of the central flowline may have enhanced the ablation due to the increased absorption of solar radiation and a shorter vertical distance for heat conduction^71^, and thus the higher observed mass loss on the right side of the central flowline. The debris layer on the left side of the central flowline, on the other hand, is comparatively thicker, with an average depth of 22 cm, resulting in the lowest measured mass loss on the left side of the HG. Because the average thickness of the debris along the central flowline of the glacier is 16 cm, stake data along the central flowline revealed a medium mass loss of − 1.35 ± 0.39 m w.e. Several studies on the impact of debris-cover on glacier recession in the Indian Himalaya have concluded that debris-cover has a substantial impact on glacier melt^13,72^, and that the thickness and distribution of debris-cover affect the pattern and degree of glacial mass balance^73^.

Differential thinning of the debris-covered and clear-ice parts of glaciers has been reported in studies and varies depending upon debris-cover distribution, lithology, pattern and thickness^74,75^. Thin debris-covered ice melts at a rate of around 9% faster than clean ice^75^. Thick debris-covered ice melts at a third of the pace of clean ice^76^. Popovnin and Rozova^77^ found that a glacier with debris thickness of 7–8 cm melts faster. Due to the absorption of incident solar radiation^71^ and the concomitant low albedo of debris-cover relative to snow and ice^78^, it has been hypothesised that thin debris-cover increases sub-debris melt rates. The findings of this study are consistent with earlier studies that suggest that glaciers with heavy debris cover have slower thinning rates and vice versa^51,71^.

### Altitudinal variation of the mass balance

Glacier mass balance varies significantly with altitude^64,72^. The mean mass balance data of the debris-covered HG (point specific) was analysed by altitude ranges; 3690–3750 m, 3750–3800 m, and 3800–3850 m at around 50 m intervals in the ablation zone and a single zone with 3850–4350 m altitude in the steep ablation and accumulation zone to understand the altitudinal variation of the observed point mass balance. From Table 3, it is evident that the ablation zone at the lower elevations (3690–3750 m) showed a mean mass loss of − 1.29 ± 0.39 m w.e (SD of 0.20) based on the average of the measurements from 6 stakes, while the ablation zone at the mid-altitudes (3750–3800 m) showed a mean mass balance of − 1.38 ± 0.39 m w.e (SD of 0.07) based on the average of the measurements from 4 stakes and the three ablation stakes in 3800–3850 m altitude zone showed a mean mass loss of − 1.44 ± 0.39 m w.e (SD of 0.12). The mean mass loss observed from one stake (because of the steep slope and highly crevassed glacierized areas in the elevation zone) and three snow pit measurements in the accumulation zone situated between 3850 and 4350 m was positive (0.51 ± 0.39 m w.e SD of 0.20). In contrast to the less negative mass balance trend with the increasing altitude that is typical of most mountain glaciers^72,79^, this study found an opposite altitudinal trend of the mass balance in the ablation zone. The presence of the extensive and thick debris cover with an average thickness of 17 cm in the lower ablation zone (3690–3750 m) that thins out gradually to 14 cm in the mid-altitude ablation zone (3750–3800 m) and 5 cm in the upper ablations zone (3800–3850 m) is the most plausible explanation for the observed mass loss increase with increasing altitude up to around the ELA. The insulating effect of the thick debris cover reduces ablation rates due to the reduced thermal conductivity of the debris cover^73^. The higher negative mass balance of − 1.44 ± 0.39 m w.e. observed at higher altitudes is explained by the presence of thinner debris coverage (~ 5 cm thick), which results in more absorption of the incident solar radiation, promoting glacier melting^21^. The intricate interplay of controlling factors for debris-covered glaciers, according to Pellicctiotti et al.^71^, defies the application of standard glaciological notions such as the elevation-wise mass-balance gradient.

**Table 3:** Altitudinal variation of mass balance of the Hoksar Glacier.

Altitude (m)	Mass balnce (m w.e.)	SD	Debris thickness (cm)
3690–3750	− 1.29	0.20	–17
3750–3800	− 1.38	0.07	–14
3800–3850	− 1.44	0.12	–5
3850–4350	0.51	0.20	–2

### Geodetic mass balance

**Figure 4:**
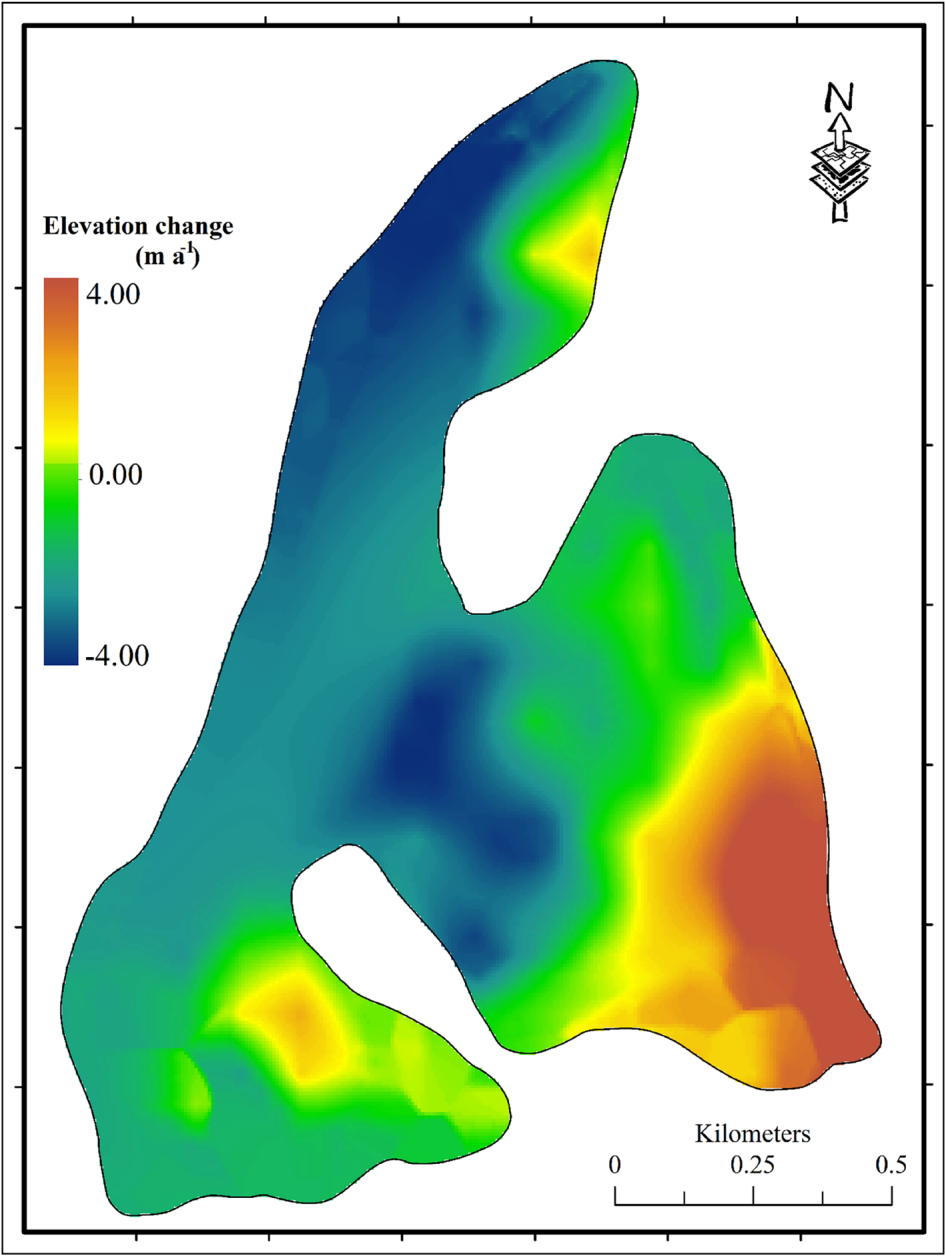
Interpolated elevation changes of the Hoksar Glacier from 2000 to 2012.

The mean geodetic mass balance estimated using the SRTM and TanDEM-X DEMs (2000–2012) was − 1.20 ± 0.35 m w.e.a^−1^. The HG thickness change varies from + 3.70 to − 3.67 m w.e.a^−1^ (Fig. 4). The glacier mass loss diminishes as it approaches the accumulation area^80^ which contradicts the direct mass loss findings. This could be attributed to the recent significant increase in the debris cover of the HG, which has increased from ~ 23% in 2001 to ~ 37% in 2016^20,81^, as observed from satellite data. The marginal mass gain was found in the lower ablation zone of the glacier, which might be explained justified by an increase in debris thickness in the lower ablation zone of the glacier during the observation period^82,83^. Thinning reaching up to around − 4 m a^−1^ on the eastern flank of the glacier can be attributed to the ice-calving at the ice-fall of the glacier. The mean geodetic mass balance (2000–2012) of the HG is broadly consistent with the glaciological mass balance (2013–2018) of − 0.95 ± 0.42 m w.e. a^−1^ observed for the Greater Himalayan range^8^. The assessment offers insight into the changes in the glacier melting pattern of the glacier from the recent past, despite the fact that geodetic and in situ mass balances are estimated for two separate time periods. The geodetic approach has been primarily used to estimate the glacial mass balance in the Kashmir Himalayan region, and the results suggest a heterogeneous behaviour of negative mass balance in the region ^4,8,13^.

### Equilibrium line altitude (ELA)

The ELA, demarcated in the field during the annual field expeditions from 2013 to 2018 showed a clear upward shift of 40 ± 3 m ranging between 4022 m in 2013 and 4062 m in 2018 with an average ELA of 4045 ± 5 m (Table 2). There is a direct relationship between mass balance and ELA changes^44,56^. The mass loss trend is followed by the up-glacier movement of the end-of-melt-season snowline, which is an approximation of ELA for Himalayan glaciers.

### Climatic influences on glacier mass balance

**Figure 5:**
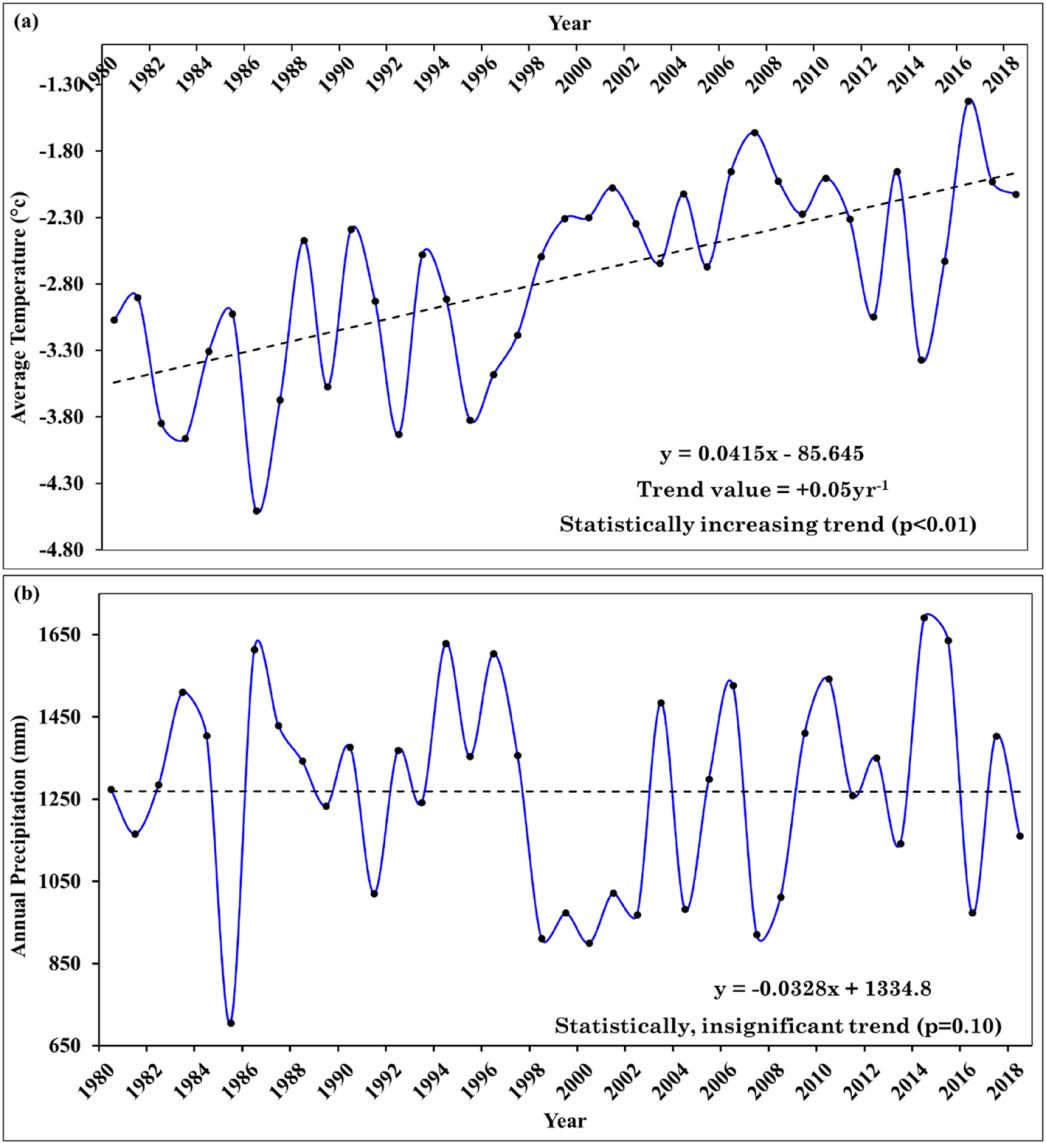
Temporal variations of (**a**) average annual temperature on the Hoksar Glacier from 1980 to 2018 calculated by using the temperature lapse-rate from the nearest observatory at Pahalgam (19 km away), and (**b**) total annual precipitation observed from 1980 to 2018 at Pahalgam.

The two key factors that control glacier mass balance are temperature and precipitation^24,84,85^. The Western Disturbances, bring precipitation in the Lidder basin, both in the form of rain and snow^86^. From October to April, snowfall is the most common form of precipitation^30^. During these 39 years, the mean annual lapse-rate-adjusted temperature of the study area showed a continuously increasing trend from 1980 to 2018 with inter-annual variations and ranged from a minimum of − 4.51 °C to a maximum of − 1.42 °C. The analysis showed that the mean annual temperature is increasing at the rate of 0.05 °C a^−1^. The lapse-rate adjusted temperature data at the HG site showed a significant increasing trend (p < 0.01) (Fig. 5a). Several studies have found a strong negative relationship between mass balance and temperature^24,85,87^. Changes in the form and amount of precipitation have an impact on glacier mass balance. Similarly, precipitation data analysis revealed that the highest precipitation of 1691 mm was observed in 2014 and the lowest of 705 mm in 1985 with an average annual precipitation of 1260 mm based on the long-term meteorological observations from 1980 to 2018. Trend analysis of the precipitation data showed that there is no statistically significant trend in total precipitation during the period (Fig. 5b). However, it has been reported that, while the total amount of precipitation has remained largely unchanged, the precipitation phase has changed from snow to rainfall as a result of the rise in the average minimum and maximum temperatures in the area^88^. During the last few decades, the amount of snowfall in the region has decreased and correspondingly, the amount of rainfall has increased^29,30^. The long-term impact of the reduced snowfall during winters in the Kashmir Himalaya has resulted in less snow accumulation over glaciers in the region, leading to a negative mass balance of glaciers in the region, as evidenced by the consistent negative mass balance of the HG from 1980 to 2018.

**Figure 6:**
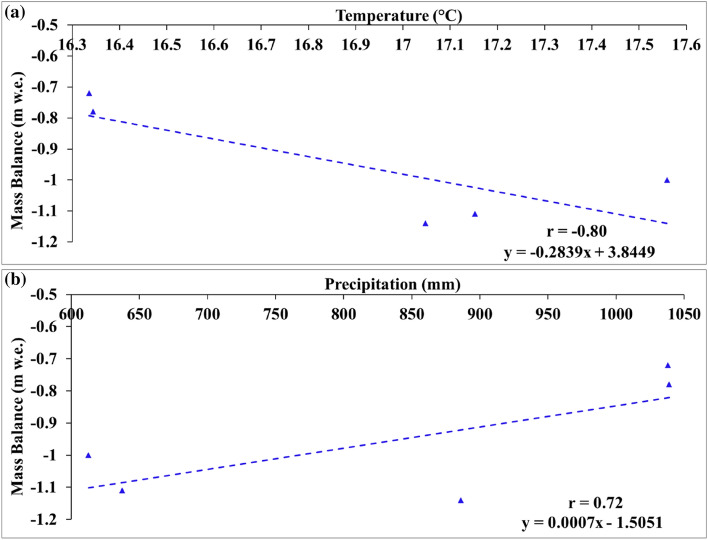
Correlation of the observed annual mass balance with (**a**) annual summer temperature and (**b**) annual winter precipitation from 2013 to 2018.

In the short term, the increasing summer temperatures and depleted snow precipitation during winters (Fig. 6a,b) is reflected in the mass balance of the HG. A direct and significant correlation was found between the observed glacier mass loss and summer temperature (r = − 0.80, p < 0.01) and winter precipitation (r = 0.72, p < 0.01) during the 5 years of the in-situ glacier mass balance measurements from 2013 to 2018.

### Black carbon impacts on glacier mass balance

**Figure 7:**
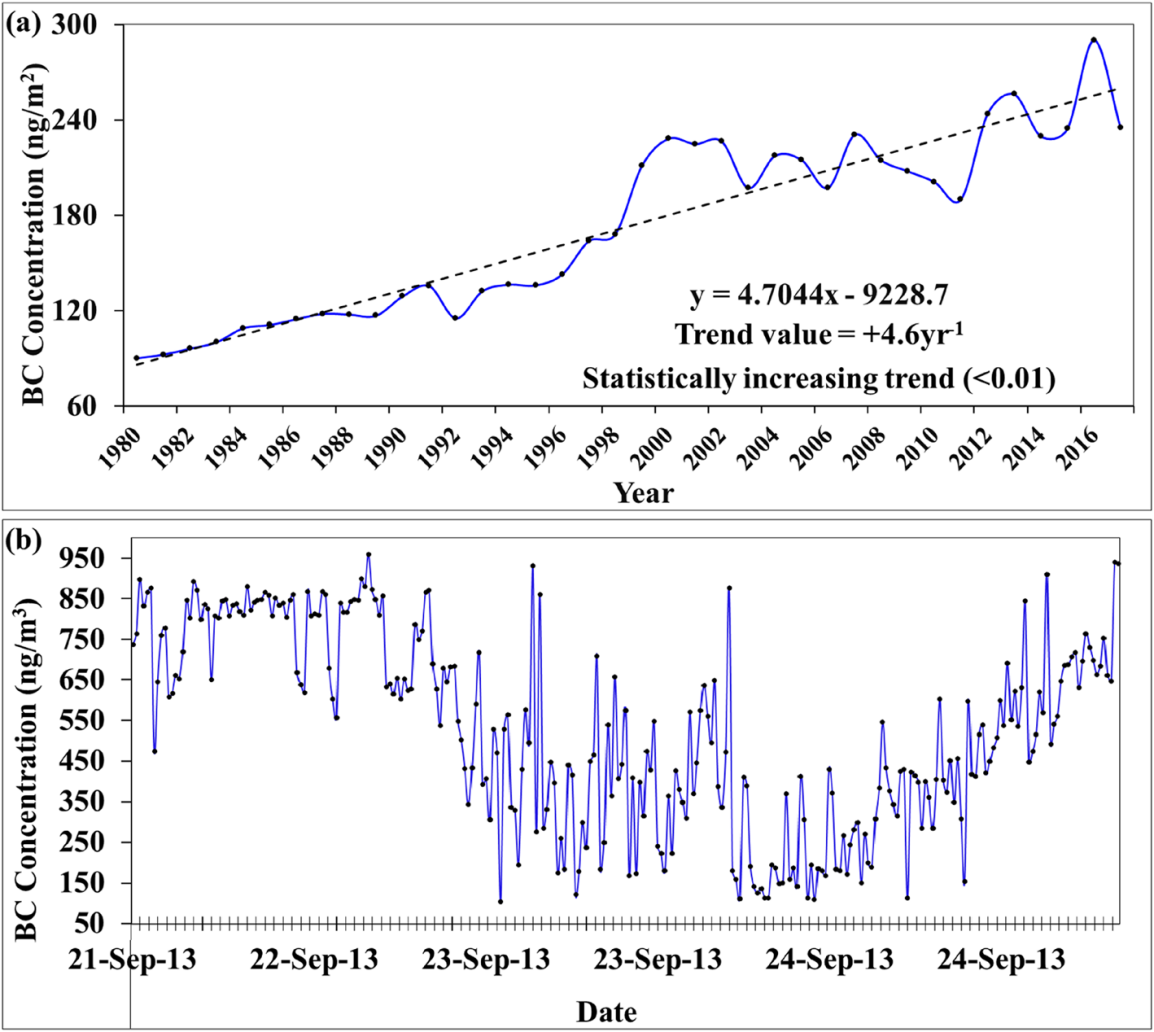
(**a**) Variation of the area-averaged MERRA BC concentration in the Lidder valley from 1980 to 2017, and (**b**) variation of the observed BC concentration at the ablation zone of the HG from 21 to 24 September, 2013.

Various studies on black carbon (BC) have found that an increase in BC concentration in the atmosphere contributes significantly to atmospheric warming since BC is a strong heat-absorbing atmospheric constituent, thus enhancing glacier ablation^5,31^. The BC concentration in the area has significantly increased from ~ 90 ng m^−2^ in 1980 to ~ 235 ng m^−2^ in 2017 (Fig. 7a). Furthermore, the analysis of the in-situ measurements of BC concentration on ablation of the glacier revealed that BC concentration varied from 105 to 959 ng m^−3^ with an average of 531 ng m^−3^ (Fig. 7b), which is significantly higher than the BC concentration reported at other glacier sites in the Himalaya^89^. It is thus inferred that the increasing trend of the BC in the valley has impacted the glacier health^20^, as is evident from the growing trend of mass loss. Bhat and others^33^ have also reported higher BC concentration over the Kashmir Himalaya and suggested its adverse impacts on glacier health. However, it needs detailed investigation to ascertain the magnitude of the impact of the increasing BC concentration over the glacier melting at a regional scale.

## Conclusions

Given the lack of knowledge about glacier mass loss in the UIB, this paper contributes significantly by offering insights into the mass balance of one of the glaciers in the UIB's Jhelum sub-basin. The two approaches used in this study produced a good agreement in MB estimations, indicating that the Hoksar Glacier is increasingly losing a significant amount of ice mass. The in situ glaciological measurements from 2013 to 2018 revealed that the mean annual mass loss of the HG is − 0.95 ± 0.39 m w.e., with an increasingly negative mass balance trend recorded during the five hydrological years. The geodetic mass balance estimates from 2000 to 2012, revealed a higher mean negative mass balance of − 1.20 ± 0.35 m w.e.a^−1^. The analysis revealed that the debris-cover had a significant impact on the glacier melting with the thick debris-covered ablation zone at lower altitudes showing less mass loss than the thin debris-covered ablation zone at higher altitudes. This shows that the regulating mechanisms for debris-covered glaciers that defy the normal elevation mass-balance gradient are complexly intertwined. The increasing mass loss of the HG has influenced its extent and health as evidenced by the glacier ELA changes, which have shifted 40 ± 3 m between 2013 and 2018, indicating the glacier imbalance. The growing negative mass balance and ascending ELA suggests that the HG is losing mass at a faster rate than the recorded mass balance estimates of the Nehnar^26^, Kolahoi and Sheshram^27^ glaciers situated in the vicinity of the glacier. Several other studies in the Jhelum have also revealed that the glaciers in the region are receding significantly^17,18^.

The significant rise in temperatures, decrease in snow precipitation and increasing concentration of black carbon in the basin, have all led to the increased mass loss of the HG. Streamflow in the basin has depleted due to the significant reduction of glacier cover and mass during the last several decades. Recent studies have suggested that glacier-fed streams saw an increase in the streamflow for a certain period of time due to the increased glacier melting, and then, exhibited a drop because of the reduction in ice mass^90,91^. However, continuing and strengthening glacier mass balance, glacio-hydrological, climatic and non-climatic data observations in the UIB is important for developing a robust understanding of glacier mass loss under changing climate and its impacts on streamflow in the UIB, so that existing gaps in knowledge are adequately addressed for developing long-term strategies for sustainable use of the depleting water resources in the UIB.

## Supplementary Information

Supplementary Information.

## Data Availability

The datasets generated or analysed during the current study are available from the corresponding author on reasonable request.

## References

[1] Molden DJ, Vaidya RA, Shrestha AB, Rasul G, Shrestha MS (2014). Water infrastructure for the Hindu Kush Himalayas. Int. J. Water Resour. Dev..

[2] Mukherji A, Sinisalo A, Nüsser M, Garrard R, Eriksson M (2019). Contributions of the cryosphere to mountain communities in the Hindu Kush Himalaya: A review. Reg. Environ. Change.

[3] Immerzeel WW, van Beek LPH, Konz M, Shrestha AB, Bierkens MFP (2012). Hydrological response to climate change in a glacierized catchment in the Himalayas. Clim. Change.

[4] Kääb A, Treichler D, Nuth C, Berthier E (2015). Brief Communication: Contending estimates of 2003–2008 glacier mass balance over the Pamir–Karakoram–Himalaya. Cryosphere.

[5] Romshoo SA (2022). Anthropogenic climate change drives melting of glaciers in the Himalaya. Environ. Sci. Pollut. Res..

[6] Azam MF (2018). Review of the status and mass changes of Himalayan–Karakoram glaciers. J. Glaciol..

[7] Bolch T (2019). Status and change of the cryosphere in the extended Hindu Kush Himalaya region. Hindu Kush Himalaya Assess..

[8] Abdullah T, Romshoo SA, Rashid I (2020). The satellite observed glacier mass changes over the Upper Indus Basin during 2000–2012. Sci. Rep..

[9] Romshoo SA, Abdullah T, Rashid I, Bahuguna IM (2022). Explaining the differential response of glaciers across different mountain ranges in the north-western Himalaya, India. Cold Regions Sci. Technol..

[10] Bhattacharya A (2021). High Mountain Asian glacier response to climate revealed by multi-temporal satellite observations since the 1960s. Nat. Commun..

[11] Vijay S, Braun M (2016). Elevation change rates of glaciers in the Lahaul-Spiti (Western Himalaya, India) during 2000–2012 and 2012–2013. Remote Sens..

[12] Raina, V. K. Himalayan glaciers: A state-of-art review of glacial studies, glacial retreat and climate change. In *Himalayan Glaciers: A State-of-Art Review of Glacial Studies, Glacial Retreat and Climate Change *(2009).

[13] Gardelle J, Berthier E, Arnaud Y, Kääb A (2013). Region-wide glacier mass balances over the Pamir-Karakoram-Himalaya during 1999–2011. Cryosphere.

[14] Quincey DJ, Glasser NF, Cook SJ, Luckman A (2015). Heterogeneity in Karakoram glacier surges. J. Geophys. Res. Earth Surf..

[15] Brun F, Berthier E, Wagnon P, Kääb A, Treichler D (2017). A spatially resolved estimate of High Mountain Asia glacier mass balances from 2000 to 2016. Nat. Geosci..

[16] Romshoo SA, Marazi A (2022). Impact of climate change on snow precipitation and streamflow in the Upper Indus Basin ending twenty-first century. Clim. Change.

[17] Romshoo SA, Fayaz M, Meraj G, Bahuguna IM (2020). Satellite-observed glacier recession in the Kashmir Himalaya, India, from 1980 to 2018. Environ. Monit. Assess..

[18] Murtaza KO, Romshoo SA (2017). Recent glacier changes in the Kashmir Alpine Recent glacier changes in the Kashmir Alpine Himalayas, India. Geocarto Int..

[19] Shekhar M (2017). Himalayan glaciers experienced significant mass loss during later phases of little ice age. Sci. Rep..

[20] Murtaza KO, Romshoo SA (2021). Applications of glacial geomorphological and lichenometric studies for reconstructing the Late Holocene glacial history of the Hoksar valley, Kashmir Himalaya, India. Geogr. Annal. Ser. A Geography.

[21] Ali I, Shukla A, Romshoo SA (2017). Assessing linkages between spatial facies changes and dimensional variations of glaciers in the upper Indus Basin, western Himalaya. Geomorphology.

[22] Liang L, Cuo L, Liu Q (2018). The energy and mass balance of a continental glacier: Dongkemadi Glacier in central Tibetan Plateau. Sci. Rep..

[23] Tak S, Keshari AK (2020). Investigating mass balance of Parvati glacier in Himalaya using satellite imagery based model. Sci. Rep..

[24] Mandal A, Ramanathan AL, Angchuk T, Soheb M, Singh VB (2016). Unsteady state of glaciers (Chhota Shigri and Hamtah) and climate in Lahaul and Spiti region, western Himalayas: A review of recent mass loss. Environ. Earth Sci..

[25] Kumar GV, Kulkarni AV, Gupta AK, Sharma P (2017). Mass balance estimation using geodetic method for glaciers in Baspa basin, Western Himalaya. Curr. Sci..

[26] Raina, V. K. & Srivastava, D. Glacier atlas of India. (2008).

[27] Kaul MN (1990). Glacial and Fluvial Geomorphology of Western Himalaya: Liddar Valley.

[28] Azam MF (2018). Review of the status and mass changes of Himalayan-Karakoram glaciers. J. Glaciol..

[29] Pepin N (2015). Elevation-dependent warming in mountain regions of the world. Nat. Clim. Change.

[30] Romshoo SA (2015). Implications of shrinking cryosphere under changing climate on the streamflows in the Lidder catchment in the Upper Indus Basin, India. Arct. Antarct. Alp. Res..

[31] Ramanathan V (2007). Warming trends in Asia amplified by brown cloud solar absorption. Nature.

[32] Menon S (2010). Black carbon aerosols and the third polar ice cap. Atmos. Chem. Phys..

[33] Bhat MA, Romshoo SA, Beig G (2017). Aerosol black carbon at an urban site-Srinagar, Northwestern Himalaya, India: Seasonality, sources, meteorology and radiative forcing. Atmos. Environ..

[34] Zhang R (2015). Quantifying sources, transport, deposition, and radiative forcing of black carbon over the Himalayas and Tibetan Plateau. Atmos. Chem. Phys..

[35] Gul C (2021). Black carbon concentration in the central Himalayas: Impact on glacier melt and potential source contribution. Environ. Pollut..

[36] Chen J (2019). Potential effect of black carbon on glacier mass balance during the past 55 years of Laohugou Glacier No 12, Western Qilian Mountains. J. Earth Sci..

[37] Akhtar M, Ahmad N, Booij MJ (2008). The impact of climate change on the water resources of Hindukush–Karakorum–Himalaya region under different glacier coverage scenarios. J. Hydrol..

[38] Immerzeel WW (2020). Importance and vulnerability of the world’s water towers. Nature.

[39] Vaux. Himalayan Glaciers: Climate Change, Water Resources, and Water Security. (2012) 10.17226/13449.

[40] Romshoo SA, Bashir J, Rashid I (2020). Twenty-first century-end climate scenario of Jammu and Kashmir Himalaya, India, using ensemble climate models. Clim. Change.

[41] Shean DE (2020). A systematic, regional assessment of high mountain Asia glacier mass balance. Front. Earth Sci..

[42] Cuffey KM, Paterson WSB (2010). The Physics of Glaciers.

[43] Fischer M, Huss M, Hoelzle M (2015). Surface elevation and mass changes of all Swiss glaciers 1980–2010. Cryosphere.

[44] Kulkarni AV (1992). Mass balance of Himalayan glaciers using AAR and ELA methods. J. Glaciol..

[45] Fountain AG, Tangborn WV (1985). The effect of glaciers on streamflow variations. Water Resour. Res..

[46] Hock R (2003). Temperature index melt modelling in mountain areas. J. Hydrol. (Amst.).

[47] Luthcke SB, Arendt AA, Rowlands DD, McCarthy JJ, Larsen CF (2008). Recent glacier mass changes in the Gulf of Alaska region from GRACE mascon solutions. J. Glaciol..

[48] Kaser G, Fountain A, Jansson P, Heucke E, Knaus M (2003). A Manual for Monitoring the Mass Balance of Mountain Glaciers.

[49] Heucke E (1999). A light portable steam-driven ice drill suitable for drilling holes in ice and firn. Geogr. Ann. Ser. B.

[50] Wagnon P (2013). Cryosphere.

[51] Vincent C (2016). Cryosphere.

[52] Cogley JG (2009). Geodetic and direct mass-balance measurements: Comparison and joint analysis. Ann. Glaciol..

[53] Nuth C, Kääb A (2011). Co-registration and bias corrections of satellite elevation data sets for quantifying glacier thickness change. Cryosphere.

[54] Huss M (2013). Density assumptions for converting geodetic glacier volume change to mass change. Cryosphere.

[55] Azam MF (2016). Meteorological conditions, seasonal and annual mass balances of Chhota Shigri Glacier, Western Himalaya, India. J. Glaciol..

[56] Benn DI, Lehmkuhl F (2000). Mass balance and equilibrium-line altitudes of glaciers in high-mountain environments. Quatern. Int..

[57] Dar RA, Jaan O, Murtaza KO, Romshoo SA (2017). Glacial-geomorphic study of the Thajwas glacier valley, Kashmir Himalayas, India. Quatern. Int..

[58] Mann HB (1945). Nonparametric tests against trend. Econom. J. Econom. Soc..

[59] Kendall, M. G. Rank correlation methods. (1948).

[60] Adnan M, Nabi G, Poomee MS, Ashraf A (2017). Snowmelt runoff prediction under changing climate in the Himalayan cryosphere: A case of Gilgit River Basin. Geosci. Front..

[61] Romshoo SA, Rafiq M, Rashid I (2018). Spatio-temporal variation of land surface temperature and temperature lapse rate over mountainous Kashmir Himalaya. J. Mt. Sci..

[62] Zemp M (2013). Reanalysing glacier mass balance measurement series. Cryosphere.

[63] Thibert E, Blanc R, Vincent C, Eckert N (2008). Glaciological and volumetric mass-balance measurements: Error analysis over 51 years for Glacier de Sarennes, French Alps. J. Glaciol..

[64] Wagnon P (2007). Four years of mass balance on Chhota Shigri Glacier, Himachal Pradesh, India, a new benchmark glacier in the western Himalaya. J. Glaciol..

[65] Kenzhebaev R (2017). Mass balance observations and reconstruction for Batysh Sook Glacier, Tien Shan, from 2004 to 2016. Cold Reg. Sci. Technol..

[66] Sold L (2016). Mass balance re-analysis of findelengletscher, Switzerland; benefits of extensive snow accumulation measurements. Front. Earth Sci..

[67] Sunako S, Fujita K, Sakai A, Kayastha RB (2019). Mass balance of Trambau Glacier, Rolwaling region, Nepal Himalaya: In-situ observations, long-term reconstruction and mass-balance sensitivity. J. Glaciol..

[68] Soheb M (2020). Mass-balance observation, reconstruction and sensitivity of Stok glacier, Ladakh region, India, between 1978 and 2019. J. Glaciol..

[69] Huber J, McNabb R, Zemp M (2020). Elevation changes of west-central Greenland glaciers from 1985 to 2012 from remote sensing. Front. Earth Sci..

[70] Vishwakarma BD (2022). Challenges in understanding the variability of the cryosphere in the Himalaya and its impact on regional water resources. Front. Water.

[71] Pellicciotti F (2015). Mass-balance changes of the debris-covered glaciers in the Langtang Himal, Nepal, from 1974 to 1999. J. Glaciol..

[72] Pratap B, Dobhal DP, Mehta M, Bhambri R (2015). Influence of debris cover and altitude on glacier surface melting: A case study on Dokriani Glacier, central Himalaya, India. Ann. Glaciol..

[73] Nicholson L, Benn DI (2006). Calculating ice melt beneath a debris layer using meteorological data. J. Glaciol..

[74] Salerno F (2017). Debris-covered glacier anomaly? Morphological factors controlling changes in the mass balance, surface area, terminus position, and snow line altitude of Himalayan glaciers. Earth Planet. Sci. Lett..

[75] Shah SS, Banerjee A, Nainwal HC, Shankar R (2019). Estimation of the total sub-debris ablation from point-scale ablation data on a debris-covered glacier. J. Glaciol..

[76] Mihalcea C (2008). Spatial distribution of debris thickness and melting from remote-sensing and meteorological data, at debris-covered Baltoro glacier, Karakoram, Pakistan. Ann. Glaciol..

[77] Popovnin VV, Rozova AV (2002). Influence of sub-Debris thawing on ablation and runoff of the Djankuat Glacier in the caucasus: Selected paper from EGS General Assembly, Nice, April-2000 (Symposium OA36). Hydrol. Res..

[78] Brock BW (2010). Meteorology and surface energy fluxes in the 2005–2007 ablation seasons at the Miage debris-covered glacier, Mont Blanc Massif, Italian Alps. J. Geophys. Res. Atmos..

[79] Dobhal DP, Thayyen R (2008). Mass balance studies of Dokriani Glacier from 1992 to 2000, Garhwal. Bull. Glaciol. Res..

[80] Oerlemans J, Reichert BK (2000). Relating glacier mass balance to meteorological data by using a seasonal sensitivity characteristic. J. Glaciol..

[81] Murtaza KO (2019). Historical Changes in the Alpine Glacier System a Case Study of Lidder Valley.

[82] Dobhal DP, Mehta M, Srivastava D (2013). Influence of debris cover on terminus retreat and mass changes of Chorabari Glacier, Garhwal region, central Himalaya, India. J. Glaciol..

[83] Anderson LS, Anderson RS (2018). Debris thickness patterns on debris-covered glaciers. Geomorphology.

[84] Oerlemans J (2005). Extracting a climate signal from 169 glacier records. Science.

[85] Zhang G, Li Z, Wang W, Wang W (2014). Rapid decrease of observed mass balance in the Urumqi Glacier No 1, Tianshan Mountains, central Asia. Quatern. Int..

[86] Zaz SN, Romshoo SA, Krishnamoorthy RT, Viswanadhapalli Y (2019). Analyses of temperature and precipitation in the Indian Jammu and Kashmir region for the 1980–2016 period: Implications for remote influence and extreme events. Atmos. Chem. Phys..

[87] Kumar R (2021). Surface mass balance analysis at Naradu Glacier, Western Himalaya, India. Sci. Rep..

[88] Shekhar MS, Chand H, Kumar S, Srinivasan K, Ganju A (2010). Climate-change studies in the western Himalaya. Ann. Glaciol..

[89] Nair VS (2013). Black carbon aerosols over the Himalayas: Direct and surface albedo forcing. Tellus B Chem. Phys. Meteorol..

[90] Thayyen RJ, Gergan JT (2010). Role of glaciers in watershed hydrology: A preliminary study of a" Himalayan catchment". Cryosphere.

[91] Marazi A, Romshoo SA (2018). Streamflow response to shrinking glaciers under changing climate in the Lidder Valley, Kashmir Himalayas. J. Mt. Sci..

