# Supplementary material for: Towards understanding various influences on mass balance of the Hoksar Glacier in the Upper Indus Basin using observations
Source: Sci Rep. 2022 Sep 19;12:15669. doi: 10.1038/s41598-022-20033-w (PMC9485142; doi:10.1038/s41598-022-20033-w)
Supplement: Supplementary file 1 — Supplementary Information. [file 41598_2022_20033_MOESM1_ESM.docx]

**Supplementary Material**

**Accuracy assessment**

**1: Geodetic mass balance**

The uncertainties in the estimates of geodetic mass balance due to the DEM differencing, radar signal penetration, uncertainty due to void fill, glacier outlines, and mass conversion were considered following the methodology of Huber et al^1^ with an additional term to account for the radar penetration error^2^, assuming that all the errors are uncorrelated and random. The signal penetration was calculated as a function of elevation and varies for each pixel/elevation band. On average, we found a signal penetration value of around 2.6 m for the study region. Furthermore, owing to the higher frequency of X band, hence lesser penetration, the bias and related uncertainty of the potential radar penetration was ignored in the present study following Huber et al^1^. The uncertainty of glacier-wide specific elevation change (*Δ_h_*) was computed as follows:

$\delta_{\Delta h}= \sqrt{{\sigma_{z}}^{2}+{\sigma_{voidfill}}^{2}+{\sigma_{TDXdate}}^{2}+{\sigma_{penatration}}^{2}}$ (6)

Where, $\sigma_{z}$, $\sigma_{TDXdate}$ and $\sigma_{penatration}$ are uncertainties in elevation change rates, TanDEM-X and radar signal penetration respectively.

Uncertainty in the elevation change rate were estimated by evaluating the off-glacier elevation changes^3^. The elevation changes were corrected for outliers by widely used Normalized Median Absolute Deviation (NMAD) approach^4^ as:

$NMAD=1.4826.median.\left[ \left| \Delta hj-m\Delta h \right| \right]$
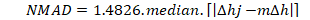
(7)

Where, Δ_hj_ and *m*Δ_h_ denotes the individual elevation changes and median of all the Δ_hj_ respectively. The influence of the outliers is significantly minimised in the NMAD approach and hence is a preferred statistical uncertainty estimate^4^.

Considering the spatial autocorrelation, the final uncertainty in elevation change rate ($\sigma_{z}$) was calculated using the widely accepted approach^3^ as:

$\sigma_{z}=\left\{ \begin{aligned} \sigma\Delta h=\sqrt{\frac{A_{Cor}}{5A}} \\ \\ \sigma\Delta h=\sigma AW, A<A_{Cor} \end{aligned} \right.,A\geq A_{Cor}$ (8)

where, σ*Δh* is the off-glacier NMAD, A is the glacier area analysed and A_cor_ = π*d*^2^, with *d* being the decorrelation length and σAW is the assessed accuracy of the elevation change rates. We assumed a mean value *d*=950 for the HG^2^. The uncertainty due to the TanDEM-X date (σ_TDXdate_) was assumed to be equal to be ±2 times the annual elevation change rate from 2000 to 2012^1^. The uncertainty in radar penetration was assumed same as the correction factor itself^1^. A constant value of ±60 kg m^-3^ was used to account for the uncertainty associated with the volume to mass conversion^5^.


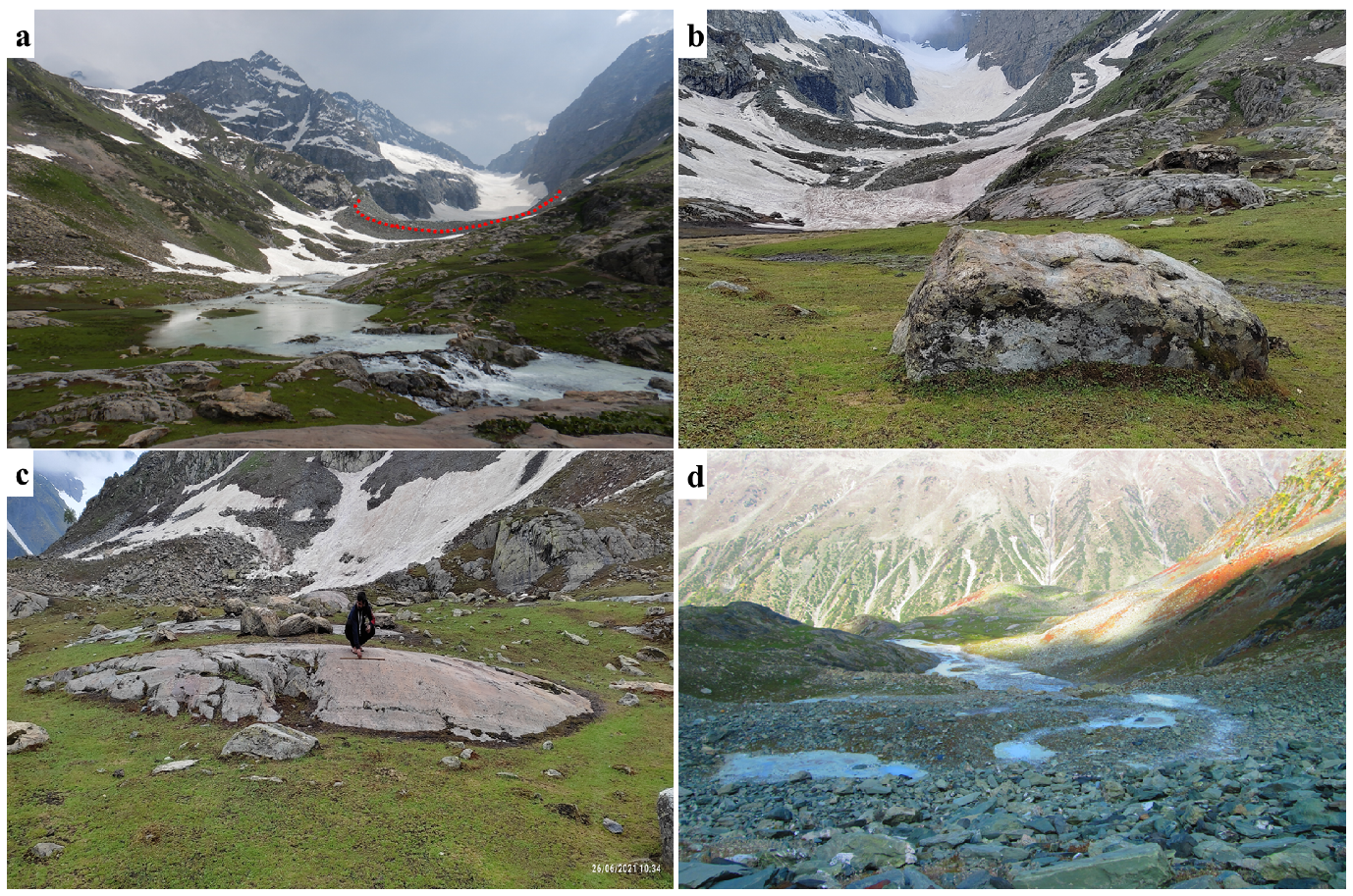


**Fig. S1.** Field photograph depicting a few prominent glacio-geomorphic landforms in the Hoksar glacier valley. (a) Horn and moraine marked with red line, (b) Erratic boulder with Hoksar Glacier in background, (c) Whaleback with striations and, (d) Outwash plain.


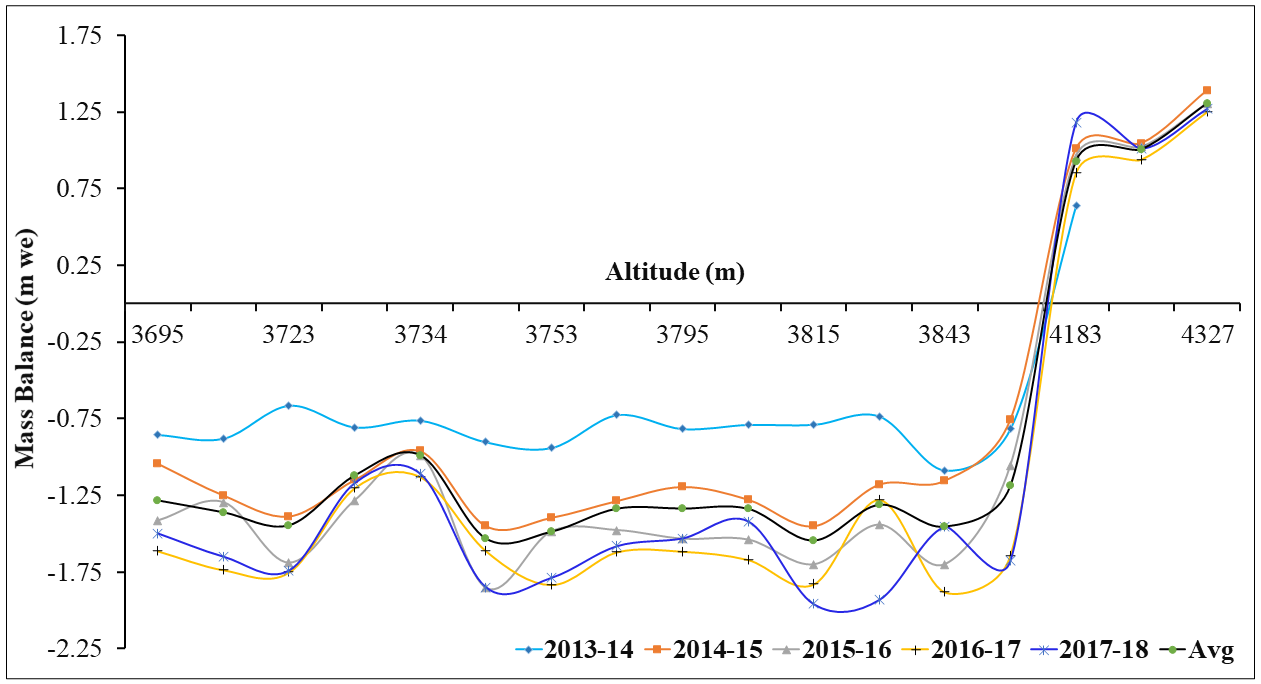


**Fig. S2.** Elevation-wise annual mass balance of the Hoksar Glacier from 2013 to 2018.

**Table S1.** Details of the glaciers observed for glaciological mass balance in the Indian Himalaya

| Glacier | Mass balance  (m w.e. a^-1)^ | Observation years | References |
| --- | --- | --- | --- |
| Hamtah | -1.43 | 2001- 2012 | Mishra and others^6^ |
| Dunagiri | -1.03 | 1984-1990 | Swaroop and Gautam^7^ |
| Hoksar | -0.95 | 2013-2018 | *Present Study* |
| Chorabari | -0.72 | 2004-2010 | Dobhal and others^8^ |
| Naradu | -0.85 | 2011-2018 | Kumar and others^9^ |
| Gor Garang | -0.57 | 1977-1985 | Kulkarni ^10^ |
| Siachen | -0.51 | 1987-1991 | Bhutiyani^11^ |
| Nehnar | -0.50 | 1976-1984 | Raina and Srivastava^12^ |
| Chhota Shigri | - 0.46 | 2002–2019 | Mandal and others^13^ |
| Shaune Garang | -0.40 | 1984-1989 | Singh and Sangewar^14^ |
| Stok | −0.39 | 2014-2019 | Soheb and others^15^ |
| Patsio | −0.34 | 2010-2019 | Angchuk and others^16^ |
| Dokriani | -0.32 | 1992-2013 | Dobhal and others^17^ |
| Gara | -0.32 | 1974-1983 | Raina and others^18^ |
| Changme Khangme | -0.29 | 1979-1986 | Raina and Srivastava^19^ |
| Shishram | -0.29 | 1983-1984 | Kaul^20^ |
| Kolahoi | -0.26 | 1983-1984 | Kaul^2019^ |
| Tipra Bank | -0.24 | 1981-1988 | Raina and Srivastava^19^ |

**References:**

1. Huber, J., McNabb, R. & Zemp, M. Elevation changes of west-central Greenland glaciers from 1985 to 2012 from remote sensing. *Frontiers in Earth Science* **8**, 35 (2020).

2. Abdullah, T., Romshoo, S. A. & Rashid, I. The satellite observed glacier mass changes over the Upper Indus Basin during 2000–2012. *Scientific Reports* **10**, (2020).

3. Seehaus, T. *et al.* Changes of the tropical glaciers throughout Peru between 2000 and 2016–mass balance and area fluctuations. *The Cryosphere* **13**, 2537–2556 (2019).

4. Höhle, J. & Höhle, M. Accuracy assessment of digital elevation models by means of robust statistical methods. *ISPRS Journal of Photogrammetry and Remote Sensing* **64**, 398–406 (2009).

5. Huss, M. The Cryosphere Density assumptions for converting geodetic glacier volume change to mass change. 877–887 (2013) doi:10.5194/tc-7-877-2013.

6. A, K. & R, S. D. M. Long term monitoring of mass balance of Hamtah Glacier, Lahaul and Spiti district, Himachal Pradesh. *Geological Survey of India.* Preprint at (2014).

7. S, S. & CK, G. Glaciological studies on Dunagiri glacier, Chamoli District, Uttar Pradesh. . *Geological Survey of India* (1990).

8. Dobhal, D. P., Mehta, M. & Srivastava, D. Influence of debris cover on terminus retreat and mass changes of Chorabari Glacier, Garhwal region, central Himalaya, India. *Journal of Glaciology* **59**, 961–971 (2013).

9. Kumar, R. *et al.* Surface mass balance analysis at Naradu Glacier, Western Himalaya, India. *Scientific Reports 2021 11:1* **11**, 1–12 (2021).

10. Kulkarni, A. v. Mass balance of Himalayan glaciers using AAR and ELA methods. *Journal of Glaciology* **38**, 101–104 (1992).

11. Bhutiyani, M. R. Mass-balance studies on Siachen glacier in the Nubra valley, Karakoram Himalaya, India. *Journal of Glaciology* **45**, 112–118 (1999).

12. VK, R. & D, S. Glacier atlas of India. Geological Society of India, Bangalore, 316. Preprint at (2008).

13. Mandal, A. *et al.* Understanding the interrelationships among mass balance, meteorology, discharge and surface velocity on Chhota Shigri Glacier over 2002–2019 using in situ measurements. *Journal of Glaciology* **66**, 727–741 (2020).

14. RK, S. & CV, S. Mass balance variation and its impact on glacier flow movement at Shaune Garang Glacier, Kinnaur, Himachal Pradesh. *Proc National Meet on Himalayan Glaciology, Department of Science and Technology, New Delhi* (1989).

15. Soheb, M. *et al.* Mass-balance observation, reconstruction and sensitivity of Stok glacier, Ladakh region, India, between 1978 and 2019. *Journal of Glaciology* **66**, 627–642 (2020).

16. Angchuk, T. *et al.* Annual and seasonal glaciological mass balance of Patsio Glacier, western Himalaya (India) from 2010 to 2017. *Journal of Glaciology* **67**, 1137–1146 (2021).

17. Dobhal, D. P., Pratap, B., Bhambri, R. & Mehta, M. Mass balance and morphological changes of Dokriani Glacier (1992–2013), Garhwal Himalaya, India. *Quaternary Science Advances* **4**, 100033 (2021).

18. Raina, V. K., Kaul, M. K. & Singh, S. Mass-balance studies of Gara Glacier. *Journal of Glaciology* **18**, 415–423 (1977).

19. Raina, V. K. & Srivastava, D. Glacier atlas of India. (2008).

20. Kaul, M. N. *Glacial and Fluvial Geomorphology of Western Himalaya: Liddar Valley*. (Concept Publishing Company, 1990).
